# Liver-specific LINC01146, a promising prognostic indicator, inhibits the malignant phenotype of hepatocellular carcinoma cells both in vitro and in vivo

**DOI:** 10.1186/s12967-021-03225-2

**Published:** 2022-01-31

**Authors:** Xiaoyun Ma, Meile Mo, Chao Tan, Jennifer Hui Juan Tan, Huishen Huang, Bihu Liu, Dongping Huang, Shun Liu, Xiaoyun Zeng, Xiaoqiang Qiu

**Affiliations:** 1 Department of Epidemiology, School of Public Health, Guangxi Medical University, Nanning, Guangxi China; 2 Department of Epidemiology, School of Public Health, Cheeloo College of Medicine, Shandong University, Jinan, Shandong China; 3 Yong Loo Lin School of Medicine, National University of Singapore, 10 Medical Dr, Singapore, Singapore; 4 Department of Sanitary Chemistry, School of Public Health, Guangxi Medical University, Nanning, Guangxi China; 5 Department of Maternal, Child and Adolescent Health, School of Public Health, Guangxi Medical University, Nanning, Guangxi China; 6 Key Laboratory of Early Prevention and Treatment for Regional High Frequency Tumor (Guangxi Medical University), Ministry of Education, Nanning, Guangxi China

**Keywords:** Hepatocellular carcinoma, LINC01146, Prognosis, Malignant phenotype, In vitro, In vivo

## Abstract

**Background:**

Long non-coding RNAs (lncRNAs) are involved in the development of hepatocellular carcinoma (HCC). We aimed to investigate the function of LINC01146 in HCC.

**Methods:**

The expression of LINC01146 in HCC tissues was explored via The Cancer Genome Atlas (TCGA) and Gene Expression Omnibus (GEO) databases and was verified using quantitative real-time polymerase chain reaction (qRT–PCR) in our HCC cohort. Kaplan–Meier analysis was used to assess the relationship between LINC01146 and the prognosis of HCC patients. Cell Counting Kit 8, colony formation assays, Transwell assays, flow cytometric assays, and tumour formation models in nude mice were conducted to reveal the effects of LINC01146 on HCC cells both in vitro and in vivo. Bioinformatic methods were used to explore the possible potential pathways of LINC01146 in HCC.

**Results:**

LINC01146 was significantly decreased in HCC tissues compared with adjacent normal tissues and was found to be related to the clinical presentations of malignancy and the poor prognosis of HCC patients. Overexpression of LINC01146 inhibited the proliferation, migration, and invasion of HCC cells in vitro, while promoting their apoptosis. In contrast, downregulation of LINC01146 exerted the opposite effects on HCC cells in vitro. In addition, overexpression of LINC01146 significantly inhibited tumour growth, while downregulation of LINC01146 promoted tumour growth in vivo. Furthermore, the coexpressed genes of LINC01146 were mainly involved in the “metabolic pathway” and “complement and coagulation cascade pathway”.

**Conclusion:**

LINC01146 expression was found to be decreased in HCC tissues and associated with the prognosis of HCC patients. It may serve as a cancer suppressor and prognostic biomarker in HCC.

**Supplementary Information:**

The online version contains supplementary material available at 10.1186/s12967-021-03225-2.

## Background

Primary liver cancer is the sixth most commonly diagnosed cancer, with an estimated 905,677 new cases and 830,180 deaths worldwide in 2020 [1]. China has the heaviest burden of primary liver cancer, with 50% of all global cases [2]. Hepatocellular carcinoma (HCC) accounts for 85–90% of primary liver cancer incidence [3]. Despite the wide scope of research on HCC diagnosis and treatment, approximately 60–70% of HCC patients have a risk of recurrence within 5 years, giving rise to poor prognosis and poor quality of life [4, 5]. Therefore, it is urgent to discover novel prognostic biomarkers and satisfactory diagnostic and therapeutic strategies.

Long non-coding RNAs (lncRNAs) have attracted widespread attention in recent years due to their important role in HCC [6–10]. An increasing number of studies has proven that lncRNAs are associated with the proliferation, migration, invasion, apoptosis, differentiation, angiogenesis, and metabolism of HCC cell lines [11–14]. For instance, LINC00205, lncRNA RHPN1-AS1, and lncRNA TMPO-AS1 directly interact with microRNAs by acting as competitive endogenous RNAs (ceRNAs) to promote the proliferation, migration, and invasion of HCC cells [15–17]. These findings are helpful in exploring the role of lncRNAs in the occurrence and metastasis of HCC and establishing a new approach for identifying lncRNAs as prognostic indicators and therapeutic targets.

In recent years, tissue-specific non-coding RNAs (ncRNAs) have shown important roles in cancer. Expression of these ncRNAs is always restricted to certain normal tissues, and they show tissue-enriched features. MiRNA-122 was the first liver-specific ncRNA discovered, and it showed inhibitory effects on HCC by reducing the metastatic ability of HCC cells in vitro and tumourigenesis and angiogenesis of HCC in vivo [18]. With respect to lncRNAs, several liver-specific lncRNAs have been identified and shown cancer suppressive effects in HCC. For example, LINC01093, a novel liver-specific lncRNA, inhibits HCC cell proliferation and metastasis in vitro and in vivo by interacting with IGF2BP1 to promote GLI1 mRNA decay [19]. Our previous studies also showed that two novel liver-specific lncRNAs, FAM99B and LINC02499, may play similar inhibitory effects on the development of HCC [20, 21]. This evidence indicate that liver-specific lncRNAs are likely to have an important role in HCC.

LINC01146 is located on chromosome 14q31.3. Based on the quantitative RNA sequencing of major human organs and tissues from the Genotype Tissue Expression Project (GTEx) database, we found that the expression of LINC01146 in normal liver tissue was more than 3 times that in any other normal tissue, showing certain tissue specificity [22]. Based on the role of liver-specific lncRNAs in the development of HCC, we hypothesized that LINC01146 may be related to the occurrence and development of HCC. In the present study, we aimed to detect the expression level of LINC01146 in HCC tissues compared with adjacent normal tissues and investigated the exact effects of LINC01146 in vitro and in vivo. Furthermore, bioinformatic methods were used to explore the possible biological processes and potential pathways of LINC01146 in HCC and provide a scientific basis for subsequent molecular mechanism studies.

## Materials and methods

### Microarray assay

Five pairs of HCC tissues and their corresponding adjacent normal tissues were used to conduct a high-throughput microarray expression profile by KangChen Biotech (KangChen Biotech Inc, Shanghai, China). The samples were labelled according to the ArraryStar RNA Flash Labelling Kit specifications, and Agilent SureHyb (Agilent Technologies, Palo Alto, Calif.) was used to conduct the hybridization experiments. The specific workflow is described in detail in the article by Guo X [23].

### The Cancer Genome Atlas (TCGA) database

RNA sequencing data and the corresponding clinical information of HCC patients were obtained from the TCGA (https://cancergenome.nih.gov/) [24] database. We used RNA expression data in the transcripts per million (TPM) and converted it into the base-2 logarithm for normalization [25]. The follow-up time and survival state of clinical information were used for Kaplan–Meier analysis. Patients without LINC01146 expression, follow-up time, or survival state were excluded.

### Gene Expression Omnibus (GEO) database

LINC01146 expression in HCC and normal control samples was searched from the GEO (http://www.ncbi.nlm.nih.gov/geo/) [26] database. The search keywords were as follows: (long non-coding RNA OR lncRNAs) AND (hepatic OR liver OR hepatocellular) AND (cancer OR carcinoma OR tumour OR neoplasm). We also set the type to “series” and the species to “human”. The inclusion criteria were as follows: (1) each chip had to contain HCC tissues and normal liver tissues; (2) the expression of LINC01146 was detected in at least three cancer tissues and normal tissues; and (3) the expression of LINC01146 data was directly available or calculable.

### Tissue samples

We collected HCC tissues and adjacent normal tissues from the Affiliated Cancer Hospital of Guangxi Medical University from January 2016 to December 2019. We included patients who were about to undergo surgical excision and had not previously received additional adjuvant therapy in our HCC cohort study. The clinical features and follow-up information of patients were also collected for the survival analysis. Each patient entering our cohort signed an informed consent form. Our research was approved by the Ethics Committee of Guangxi Medical University.

### HCC cell lines and animals

In total, six HCC cell lines were used to detect the expression of LINC01146, including Huh7, Hep3B, HCCLM3, MHCC97H, SNU423, and SNU449. Dulbecco’s modified Eagle’s medium (DMEM), minimum essential medium (MEM), and Roswell Park Memorial Institute 1640 (RPMI-1640) (Gibco, USA) were utilized for cell culture. All HCC cell lines were cultured in a 37 °C incubator containing 5% CO_2_.

Twenty-four specific pathogen-free (SPF) 4-week-old male nude mice (BALB/c) were housed in the Guangxi Medical University Laboratory Animal Center. The nude mice were randomly divided into 4 groups according to their weight, with 6 mice in each group. All experimental procedures were reviewed and approved by the Ethics Committee of Guangxi Medical University, and all animal experiments were performed in the Guangxi Medical University Laboratory Animal Center [SYXKGUI 2020-0004] in accordance with the welfare and ethical standards of animal experiments in China.

### Quantitative real-time polymerase chain reaction (qRT-PCR)

Total RNA of HCC tissues and cell lines was extracted by TRIzol (Invitrogen, USA). Reverse transcription of complementary deoxyribose nucleic acid (cDNA) was conducted according to the PrimeScript RT reagent Kit (Takara, Japan). qRT–PCR was performed using the TB Green TM Premix Ex Taq TM II Kit (Takara, Japan). The primers were as follows: GAPDH forward: 5′-AGCCACATCGCTCAGACAC-3′, GAPDH reverse: 5′-GCCCAATACGACCAAATCC-3′; LINC01146 forward: 5′-TTGAAGGCAGTATGCTTGGTAA-3′, LINC01146 reverse: 5′-TTCCGCAGTGTATCGTGTCC-3′.

### Cell transfection

MHCC97H and Huh7 cells were selected to construct LINC01146 stably overexpressing cell lines through lentiviral transfection, while Huh7 and Hep3B cells were selected to construct LINC01146 stably downregulated cell lines through lentiviral transfection (Genepharma, Shanghai, China). The Lv-NC and sh-NC groups served as the negative controls of the overexpression and downregulation HCC cell lines, respectively. After seventy-two hours of lentivirus infection, we used puromycin (2.5 or 3.5 μg/ml) to screen successfully transfected HCC cells. Finally, qRT-PCR was performed to detect the efficiency of overexpression and downregulation of LINC01146 in HCC cell lines. The sequences of sh-NC and sh-LINC01146 were as follows: LINC01146-Homo-NC: 5′-TTCTCCGAACGTGTCACGT-3′; LINC01146-Homo-330: 5′-GGTCTCCAGCTTCGTCAATGT-3′.

### Cell proliferation assays

Cell Counting Kit-8 (CCK-8; Dojindo, Japan) was used to investigate the effect of LINC01146 on the proliferation ability of HCC cells. The cells were diluted to 10^4^ cells/ml and seeded into five 96-well plates. Then, we added a mixture of CCK-8 and complete medium (CCK-8: complete medium = 1:10) to each well after 24, 48, 72, 96, and 120 h of incubation. Finally, the absorbance value (OD = 450 nm) of each group was measured with a microplate reader after incubation for another 2 h.

Colony formation assays were conducted to investigate the effect of LINC01146 on the proliferation ability of HCC cells. The cells were diluted to a density of 250 cells/ml and cultured in a six-well plate until most clones had more than 50 cells. Afterwards, the cells were fixed with methanol and stained with 0.1% crystal violet for another 30 min. Finally, the treated cells were scanned, and the number of clones in each group was calculated as the average number in three parallel wells.

### Transwell assays

Transwell chambers with 8 μm pore filters (Costar, Corning, NY) were utilized to explore the effect of LINC01146 on the migration and invasion abilities of HCC cells. The specific experimental procedure was described in our previous article [21]. The cells with serum-free medium were seeded into the upper chambers, and complete media with 20% serum was added to the lower chambers. After 48 or 72 h, the cells were fixed with methanol and stained with 0.1% crystal violet, followed by observation and counting under an inverted microscope.

### Flow cytometric assays

Flow cytometry was performed to assess the cell cycle distribution in different groups. Huh7 cells were digested and washed with PBS twice when the density of cells reached 80–90% in six-well plates. Then, the cells were fixed with cold 75% ethanol overnight at 4 °C. After centrifugation, the fixed cells were washed with precooled PBS and then with periodic reagents. Afterwards, the cells were incubated with PI/RNase Staining Buffer (BD Pharmingen™, USA) at room temperature for 15 min, and the cycle distribution was immediately measured by flow cytometry.

To detect the apoptotic rate, the cells were digested by ethylenediaminetetraacetic acid (EDTA)-free trypsin, washed with precooled PBS and then with 1× buffer. The cells were later double stained with PE Annexin V and 7-AAD according to the instructions of the PE Annexin V Apoptosis Detection Kit I (BD Pharmingen™, USA). The cell apoptosis rate was analysed by flow cytometry within 1 h.

### Tumour formation model in nude mice

In total, 2 × 10^7^ MHCC97H cells and 5 × 10^7^ Huh7 cells were suspended in a 2 ml mixture (1:1, v/v) of serum-free medium and Matrigel (Costar, Corning, NY). Then, 0.2 ml of cell suspension was injected subcutaneously into the left axilla of the nude mice. The formed tumour was measured with a Vernier caliper every 5 days, and the tumour volume was calculated as follows: V = 1/2ab^2^, where V represents the volume of the tumour, while a and b represent the longest and shortest diameter of the tumour, respectively. Thirty and 25 days after the inoculation of MHCC97H and Huh7 cells, respectively, the nude mice were sacrificed by cervical dislocation, followed by removal, weighing, and measurement of the tumour tissues. Each tumour was then placed in 4% paraformaldehyde for fixation for 24 h.

### Haematoxylin–eosin (HE) staining and immunohistochemical (IHC) staining

For HE, after deparaffinization and rehydration, the sections were stained with haematoxylin for 8 min followed by 5 dips in 1% acid ethanol (1% HCl in 70% ethanol) and then rinsed in distilled water. Then, the sections were stained with 1% eosin aqueous solution for 4 min followed by dehydration with graded alcohol and clearing in xylene [27]. Finally, the sections were sealed with neutral adhesive and observed under a microscope.

For IHC, after incubating with antigen retrieval solution and 3% H_2_O_2_ for 15–20 min, the slides were rinsed with water and incubated with the primary antibody Ki-67 (Abcam, Cambridge, MA; 1:200) for 1 h at 37 °C. Then, the slides were incubated with the biotinylated secondary antibody and DAB followed by haematoxylin staining [28]. Finally, the slices were dried and sealed with neutral gum. The results were analysed by Image-Pro Plus 6.0 software.

### Coexpressed genes related to LINC01146

GEPIA (http://gepia.cancer-pku.cn) [29], MEM (https://biit.cs.ut.ee/mem/) [30], and TANRIC (https://ibl.mdanderson.org/tanric/_design/basic/index.html) [31] were used to predict the coexpressed genes of LINC01146. A Venn diagram (http://jvenn.toulouse.inra.fr/app/example.html) was generated to obtain the most promising coexpressed genes. Genes with at least two intersections in the Venn diagram were selected for further analysis.

### LINC01146 pathway analysis

To reveal the basic molecular mechanisms of LINC01146 in HCC, we conducted a pathway enrichment analysis on the coexpressed genes of LINC01146. WebGestalt (http://www.webgestalt.org/) [32] was used to conduct Gene Ontology (GO) functional annotation, and KOBAS 3.0 (http://kobas.cbi.pku.edu.cn/kobas3) [33] was utilized to construct Kyoto Encyclopedia of Genes and Genomes (KEGG) pathway enrichment. The STRING (http://string.embl.de/) [34] database was applied to construct a protein–protein interaction (PPI) network.

### Statistical analysis

The median value was used as the cut-off value to distinguish between high- and low-expression groups, and Student’s *t test* was performed to compare the expression levels of LINC01146 in HCC tissues and normal tissues. The chi-square test was performed to explore the relationship between LINC01146 expression and the clinical features of HCC patients. Kaplan–Meier analysis and the log-rank test were used to assess the association between LINC01146 expression and the overall survival (OS) time of HCC patients. Cox regression models were utilized to explore the independent prognostic risk factors for HCC patients. The above statistical analyses were all conducted using SPSS 22.0 software, and *P* < 0.05 was considered statistically significant. GraphPad Prism 8.0 was used to draw graphs.

Meta-analysis was conducted to integrate the GEO chip results using STATA 13.0 software. The chi-squared and *I*^2^ tests were used to explore the heterogeneity between studies. When *I*^2^ < 50% or *P* > 0.1, no significant heterogeneity existed among studies, and the fixed effect model was used for meta-analysis. Otherwise, the random-effect model was selected. A forest plot was utilized to obtain the combined effect value, including the standard mean deviation (SMD) and 95% confidence interval (CI). When SMD < 0 and *P* < 0.05, the expression of LINC01146 was considered downregulated in HCC tissues compared with adjacent normal tissues. Otherwise, the expression of LINC01146 was considered upregulated or unaltered in HCC tissues compared with adjacent normal tissues. Sensitivity analysis was used to evaluate the robustness of the meta-analysis results. The funnel graph and Egger’s test were applied to evaluate reporting bias, with *P* > 0.1 deemed to indicate no apparent reporting bias.

## Results

### LINC01146 is downregulated based on lncRNA microarray, TCGA and GEO databases

In our study, under the restriction of fold change (FC) > 2 and adjusted *P* < 0.05, we found 4433 differentially expressed lncRNAs in HCC, including 1708 upregulated lncRNAs and 2725 downregulated lncRNAs (GSE93789, Additional file 1: Fig. S1A). The GTEx database showed that LINC01146 was only expressed in a few human normal tissues and was highly expressed in normal liver tissues, which exhibited liver-specific expression characteristics (Additional file 1: Fig. S1B). Hence, we selected liver-specific LINC01146, which was downregulated in HCC tissues, as our research object (*FC* = 3.92, *P* = 6.93E−4, *FDR* = 0.027; Additional file 2: Table S1).

**Fig. 1:**
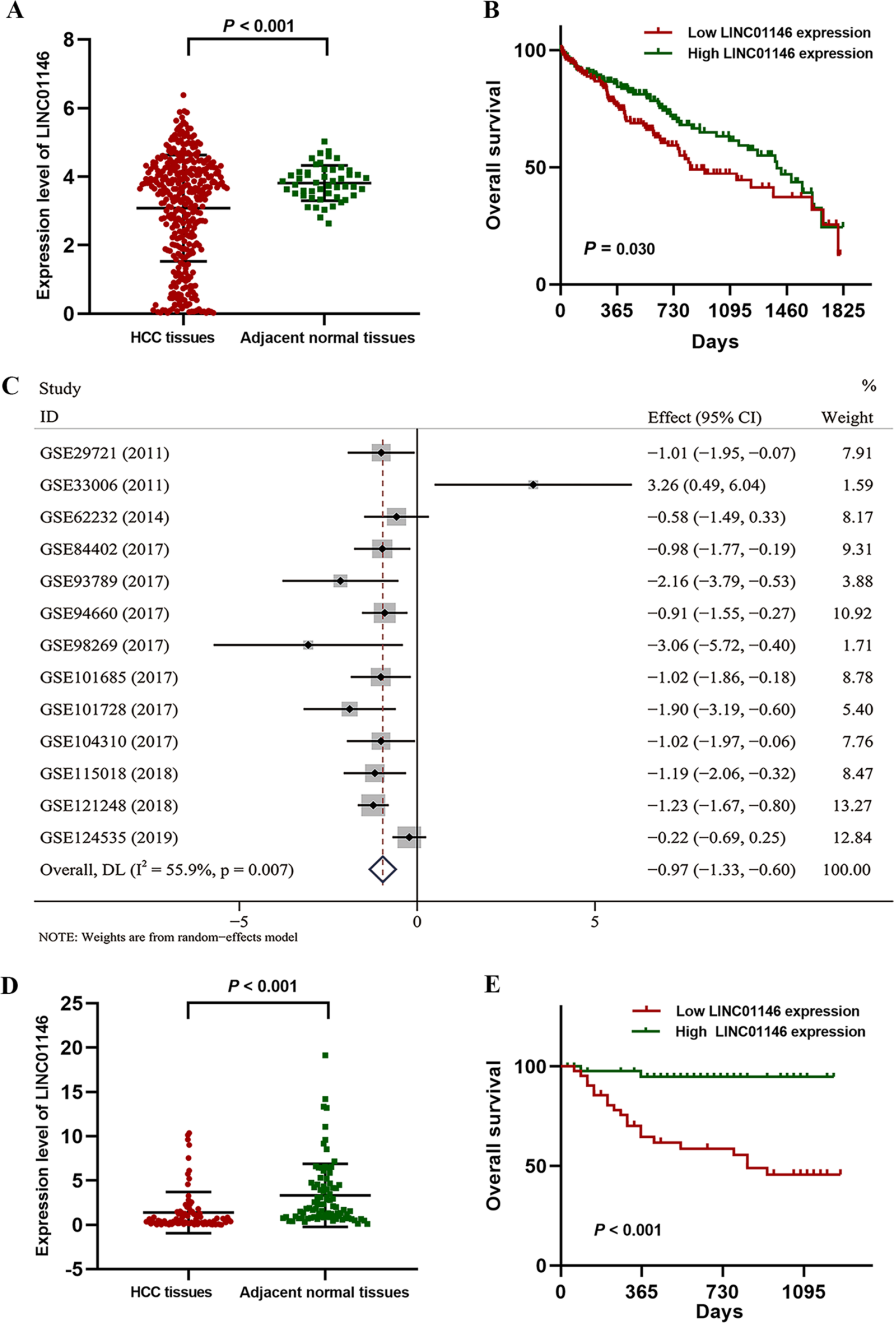
The expression level of LINC01146 in HCC tissues. **A** LINC01146 was downregulated in HCC tissues based on the TCGA database. **B** The expression of LINC01146 was associated with the 5-year overall survival of HCC patients based on the TCGA database. **C** LINC01146 was decreased in HCC tissues based on GEO datasets (with the random-effect model). **D** LINC01146 was downregulated in HCC tissues from Guangxi HCC patients (n = 88). **E** Low expression of LINC01146 was associated with poor overall survival in Guangxi HCC patients (n = 85)

For the TCGA database, the results showed that LINC01146 was decreased in HCC tissues compared with normal liver tissues (*P* < 0.001; Fig. 1A). In total, 330 HCC patients with complete follow-up information were used for 5-year OS analysis. The results indicated that low LINC01146 expression was associated with poor 5-year OS in HCC patients (*P* = 0.030; Fig. 1B).

Overall, thirteen chips were involved in the meta-analysis, and detailed information is presented in Table 1. We selected the random-effect model due to heterogeneity among these studies (*P* = 0.007; Fig. 1C). The forest plot showed that LINC01146 was decreased in HCC tissues compared with normal liver tissues (SMD = − 0.97, 95% CI [− 1.33, − 0.60], *P* < 0.001; Fig. 1C). Moreover, the results showed that no reporting bias was discovered in our study (*P* = 0.656; Additional file 1: Fig. S1C). The sensitivity analysis also showed that the results of this meta-analysis were stable (Additional file 1: Fig. S1D).

**Table 1:** The basic information and expression of LINC01146 in the GEO datasets included in this study

GEO datasets	Year	Platform	Country	Tissue types	N	LINC01146 expression (χ ± s)	*t*	*P* value
GSE29721	2011	GPL570	Canada	HCC tissue	10	3.85 ± 1.42	2.21	0.054
				Normal tissue	10	4.88 ± 0.24		
GSE33006	2011	GPL570	China	HCC tissue	3	8.51 ± 0.14	3.70	0.066
				Normal tissue	3	7.51 ± 0.41		
GSE62232	2014	GPL570	France	HCC tissue	81	1.39 ± 0.37	2.90	0.020
				Normal tissue	5	1.60 ± 0.14		
GSE84402	2017	GPL570	China	HCC tissue	14	1.17 ± 0.47	2.78	0.016
				Normal tissue	14	1.53 ± 0.22		
GSE93789	2017	GPL16956	China	HCC tissue	5	4.69 ± 2.65	6.86	< 0.001
				Normal tissue	5	8.77 ± 0.37		
GSE94660	2017	GPL16791	USA	HCC tissue	21	1.96 ± 1.08	3.10	0.003
				Normal tissue	21	2.69 ± 0.34		
GSE98269	2017	GPL20712	China	HCC tissue	3	7.12 ± 0.56	3.70	0.066
				Normal tissue	3	8.37 ± 0.14		
GSE101685	2017	GPL570	China	HCC tissue	24	4.75 ± 1.12	2.51	0.018
				Normal tissue	8	5.77 ± 0.38		
GSE101728	2017	GPL21047	China	HCC tissue	7	7.92 ± 0.94	3.33	0.016
				Normal tissue	7	9.24 ± 0.29		
GSE104310	2017	GPL16791	China	HCC tissue	12	5.88 ± 3.42	2.23	0.038
				Normal tissue	8	9.09 ± 2.68		
GSE115018	2018	GPL20115	China	HCC tissue	12	1.87 ± 1.74	3.12	0.010
				Normal tissue	12	3.41 ± 0.57		
GSE121248	2018	GPL570	Singapore	HCC tissue	70	5.74 ± 1.08	7.49	< 0.001
				Normal tissue	37	6.87 ± 0.46		
GSE124535	2019	GPL20795	China	HCC tissue	35	3.97 ± 1.75	0.95	0.350
				Normal tissue	35	4.26 ± 0.62		

### Decreased expression of LINC01146 is associated with aggressive clinical features and poor prognosis of HCC patients

The qRT–PCR results indicated that LINC01146 was downregulated in HCC tissues compared with normal tissues (*P* < 0.001, n = 88; Fig. 1D). Furthermore, the expression of LINC01146 had a distinct negative association with tumour size (*P* = 0.005), tumour number (*P* = 0.020), microvascular invasion (MVI) (*P* = 0.033), satellite nodules (*P* = 0.007), DNA content of HBV (*P* = 0.018), and Barcelona Clinic Liver Cancer (BCLC) grade (*P* = 0.010), as shown in Table 2.

**Table 2:** The relationship between LINC01146 expression and clinical features of HCC patients

Characteristics	Number (n = 88)	LINC01146 levels		*χ*^*2*^	*P* value
		Low expression	High expression		
Ages (years)				0.97	0.325
≥ 50	66	31	35		
< 50	22	13	9		
Sex				0.09	0.764
Male	75	37	38		
Female	13	8	5		
Liver cirrhosis				0.77	0.381
Negative	34	15	19		
Positive	54	29	25		
Alpha fetoprotein				3.14	0.076
≤ 400 ng/ml	32	12	20		
> 400 ng/ml	56	32	24		
Tumour size				8.02	0.005
< 5 cm	35	11	24		
≥ 5 cm	53	33	20		
Tumour number				5.44	0.020
Single	74	33	41		
Multiple	14	11	3		
Microvascular invasion				4.55	0.033
Negative	45	17	27		
Positive	43	27	17		
Satellite nodules				7.31	0.007
Negative	75	33	42		
Positive	13	11	2		
Distant metastasis				0.30	0.584
Negative	85	43	42		
Positive	3	2	1		
Lymphatic metastasis				0.16	0.694
Negative	81	41	40		
Positive	7	3	4		
DNA content of HBV				5.57	0.018
Negative	49	19	30		
Positive	39	25	14		
Edmondson-Steiner grade				1.25	0.265
I + II	31	13	18		
III + IV	57	31	26		
Barcelona clinic liver cancer grade (BCLC)				9.19	0.010
A	44	15	29		
B	18	11	7		
C	26	18	8		

The Kaplan–Meier analysis indicated that decreased LINC01146 was significantly associated with poor prognosis of HCC patients (*P* < 0.001, n = 85; Fig. 1E). Moreover, the multivariate Cox regression model showed that high expression of LINC01146 was an independent protective factor for the prognosis of HCC patients (HR = 0.38, 95% CI 0.16–0.92, *P* = 0.033), as shown in Table 3.

**Table 3:** Univariate and multivariate Cox’s proportional risk model for overall survival of HCC patients (n = 85)

Variables	Univariate analysis				Multivariable analysis			
	*β*	HR	95%CI	*P* value	*β*	HR	95%CI	*P* value
Age (≥ 50 vs. < 50)	0.03	1.03	0.99–1.06	0.135				
Sex (male vs. female)	− 0.11	0.89	0.31–2.63	0.837				
Liver cirrhosis (positive vs. negative)	0.13	1.14	0.49–2.67	0.757				
AFP (> 400 ng/ml vs. ≤ 400 ng/ml )	0.93	2.54	0.87–7.45	0.090				
Tumour size (≥ 5 cm vs. < 5 cm)	0.81	2.25	0.89–5.69	0.087				
Tumour number (multiple vs. single)	0.12	1.13	0.39–3.31	0.822				
Distant metastasis (positive vs. negative)	1.23	3.42	0.44–26.73	0.242				
Lymphatic metastasis (positive vs. negative)	0.61	1.85	0.55–6.21	0.323				
DNA content of HBV (positive vs. negative)	0.12	1.13	0.50–2.53	0.768				
Satellite nodules (positive vs. negative)	1.35	3.86	1.59–9.40	0.003	0.92	2.51	0.78–8.08	0.124
BCLC grade (A vs. B vs. C)	0.42	1.53	0.98–2.38	0.061	0.01	1.01	0.57–1.79	0.983
MVI (positive vs. negative)	0.89	2.43	1.46–4.04	0.001	2.04	7.73	2.24–26.72	0.001
Edmondson-Steiner grade (> II vs. I–II)	1.91	6.73	1.58–28.63	0.010	2.36	10.57	2.14–52.15	0.004
LINC01146 expression (high vs. low)	− 1.32	0.27	0.09–0.78	0.015	− 0.97	0.38	0.16–0.92	0.033

*vs.* versus, *AFP* alpha fetoprotein, *BCLC*: Barcelona clinic liver cancer grade, *MVI* microvascular invasion

### LINC01146 inhibits the proliferation of HCC cells in vitro

**Fig. 2:**
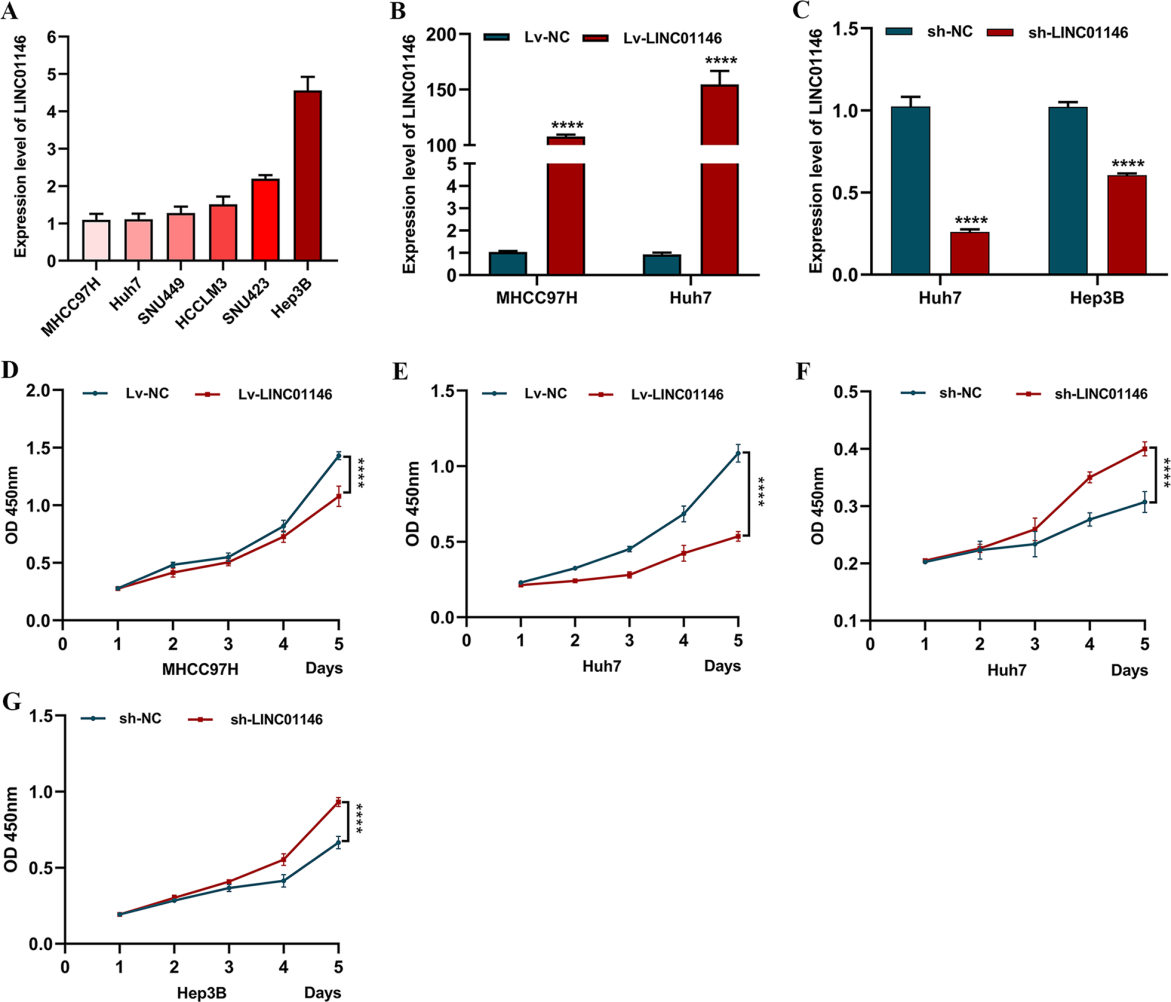
The effect of LINC01146 on the proliferation of HCC cells in vitro. **A** The expression of LINC01146 in six HCC cell lines. **B** Overexpression of LINC01146 was successfully achieved in MHCC97H and Huh7 cells. **C** Downregulation of LINC01146 was successfully achieved in Huh7 and Hep3B cells. **D**, **E** Overexpression of LINC01146 inhibited the proliferation of MHCC97H and Huh7 cells. **F**, **G** Downregulation of LINC01146 promoted the proliferation of Huh7 and Hep3B cells. ****P < 0.001

The expression of LINC01146 was lowest in MHCC97H and Huh7 cells and highest in Hep3B cells by qRT-PCR (Fig. 2A). Therefore, we chose MHCC97H and Huh7 cells to construct stable overexpressing HCC cell lines and Huh7 and Hep3B cells to construct stable knockdown HCC cell lines through lentiviral transfection. The results showed that LINC01146 was successfully overexpressed in MHCC97H (*P* < 0.001) and Huh7 cells (*P* < 0.001; Fig. 2B) but was downregulated in Huh7 (*P* < 0.001) and Hep3B cells (*P* < 0.001; Fig. 2C).

The results of CCK-8 assays indicated that the overexpression of LINC01146 inhibited the growth rate of MHCC97H (*P* < 0.001; Fig. 2D) and Huh7 cells (*P* < 0.001; Fig. 2E). In contrast, downregulation of LINC01146 increased the growth rate of Huh7 (*P* < 0.001; Fig. 2F) and Hep3B cells (*P* < 0.001; Fig. 2G).

**Fig. 3:**
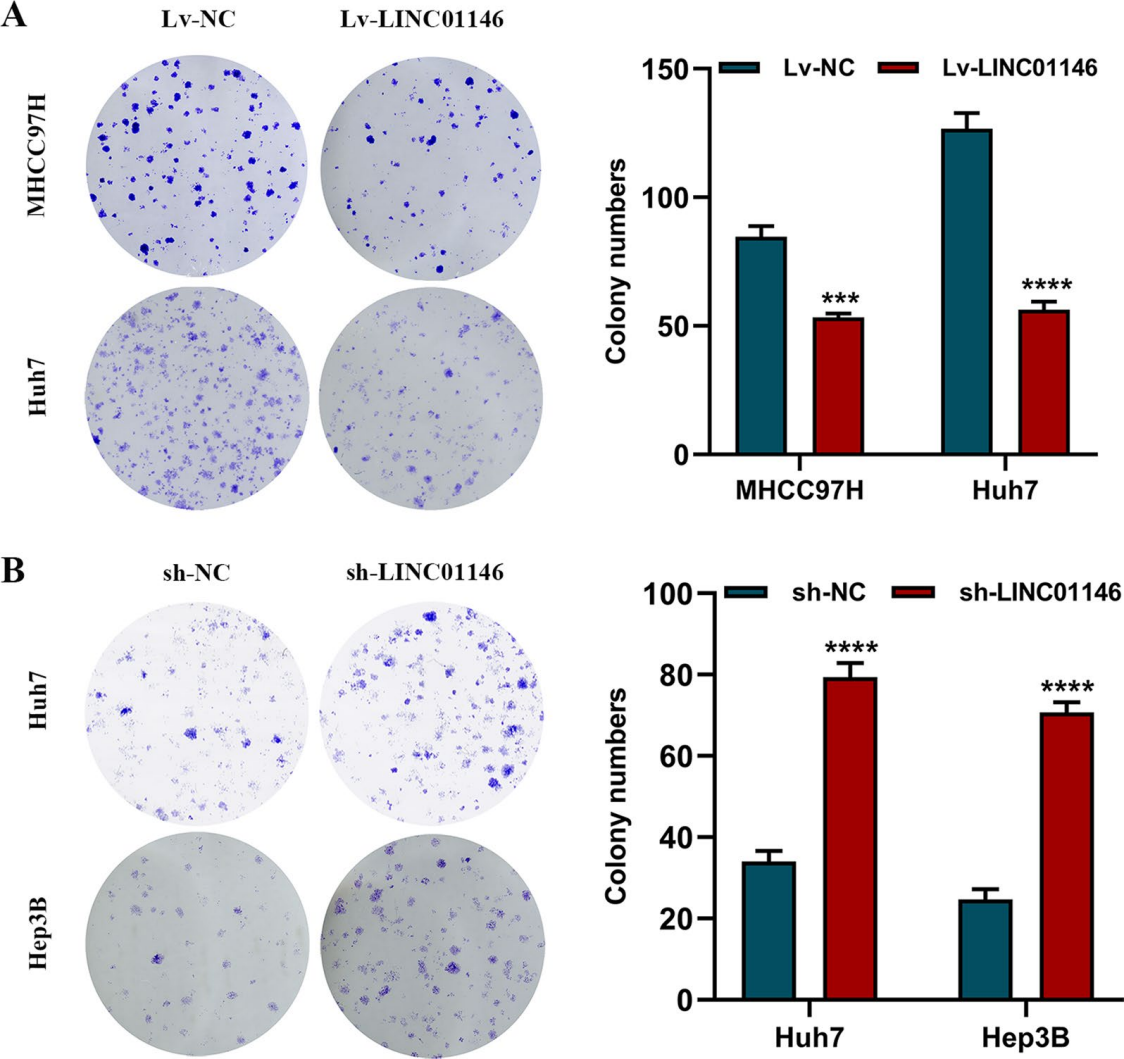
LINC01146 affects the clone formation ability of HCC cells in vitro. **A** Overexpression of LINC01146 inhibited the clone formation abilities of MHCC97H and Huh7 cells. **B** Downregulation of LINC01146 promoted the clone formation abilities of Huh7 and Hep3B cells. ***P < 0.01; ****P < 0.001

In addition, colony formation assays showed that the overexpression of LINC01146 reduced the number of colonies in MHCC97H (*P* < 0.001) and Huh7 cells (*P* < 0.001; Fig. 3A), while downregulation of LINC01146 increased the number of colonies in Huh7 (*P* < 0.001) and Hep3B cells (*P* < 0.001; Fig. 3B). All of the above results showed that LINC01146 inhibits the proliferation of HCC cell lines.

### LINC01146 inhibits the migration and invasion abilities of HCC cell lines in vitro

**Fig. 4:**
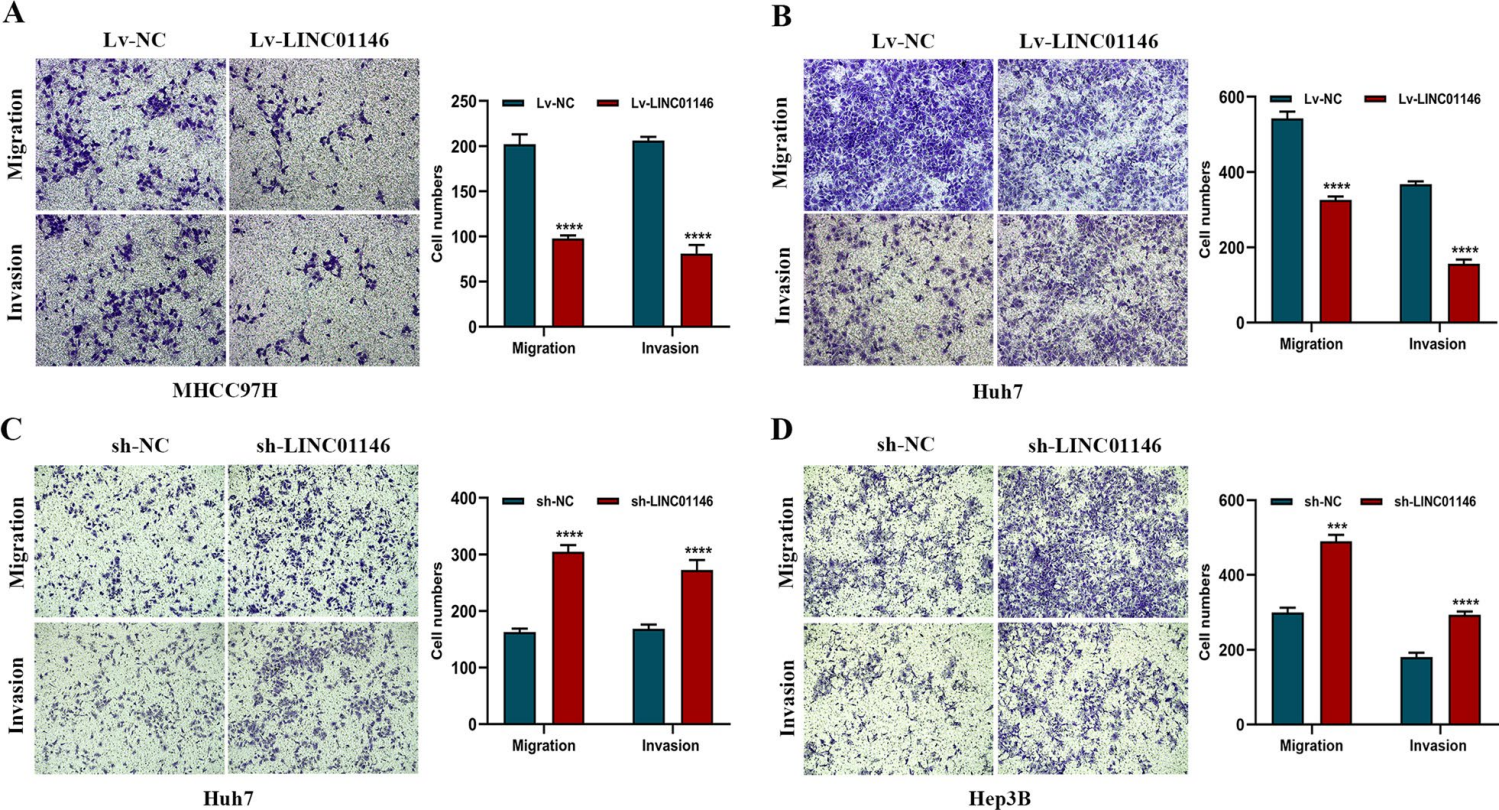
LINC01146 affects the migration and invasion abilities of HCC cells in vitro. **A** Overexpression of LINC01146 inhibited the migration and invasion of MHCC97H cells. **B** Overexpression of LINC01146 inhibited the migration and invasion of Huh7 cells. **C** Downregulation of LINC01146 promoted the migration and invasion of Huh7 cells. **D** Downregulation of LINC01146 promoted the migration and invasion of Hep3B cells. ***P < 0.01; ****P < 0.001

Transwell assays showed that overexpression of LINC01146 suppressed the migration (*P* < 0.001) and invasion (*P* < 0.001; Fig. 4A) activities of MHCC97H cells, and inhibited the migration (*P* < 0.001) and invasion (*P* < 0.001; Fig. 4B) activities of Huh7 cells. In contrast, downregulation of LINC01146 promoted the migration (*P* < 0.001) and invasion (*P* = 0.001; Fig. 4C) activities of Huh7 cells, and enhanced the migration (*P* = 0.006) and invasion (*P* < 0.001; Fig. 4D) activities of Hep3B cells.

### LINC01146 affects the cell cycle and promotes the apoptosis of HCC cells in vitro

**Fig. 5:**
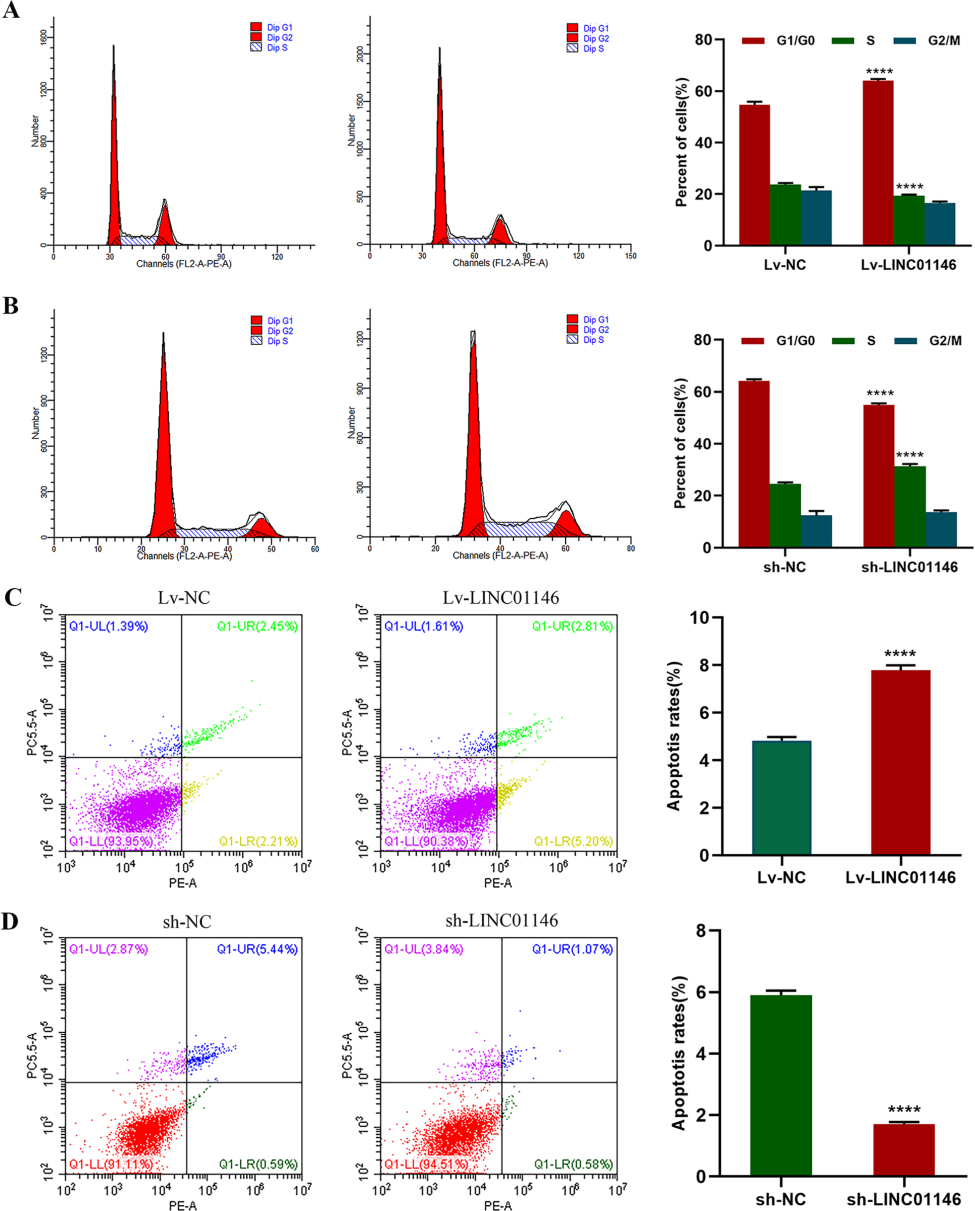
LINC01146 affects the cell cycle progression and apoptosis of HCC cells in vitro. **A** Overexpression of LINC01146 inhibited the cycle progression of Huh7 cells. **B** Downregulation of LINC01146 promoted the cycle progression of Huh7 cells. **C** Overexpression of LINC01146 promoted the apoptosis of Huh7 cells. **D** Downregulation of LINC01146 inhibited the apoptosis of Huh7 cells. ****P < 0.001

The cell cycle results showed that overexpression of LINC01146 increased the proportion of Huh7 cells in the G0/G1 phase (64.25% vs. 54.3%, *P* < 0.001), while reducing the proportion of cells in the S phase (18.95% vs. 24.21%, *P* < 0.001, Fig. 5A). In contrast, downregulation of LINC01146 reduced the proportion of Huh7 cells in the G0/G1 phase (57.59% vs. 63.99%, *P* < 0.001), while increasing the proportion of cells in the S phase (32.12% vs. 24.12%, *P* < 0.001, Fig. 5B). These results suggest that LINC01146 inhibits HCC cell proliferation by affecting the progression of the cell cycle.

Furthermore, we assessed the effect of LINC01146 on the apoptosis of HCC cells by flow cytometry. The results showed that overexpression of LINC01146 increased the apoptotic rates of Huh7 cells (*P* < 0.001, Fig. 5C), while downregulation of LINC01146 inhibited the apoptotic rates of Huh7 cells (*P* < 0.001, Fig. 5D).

### LINC01146 inhibits the tumour growth of HCC cells in vivo

**Fig. 6:**
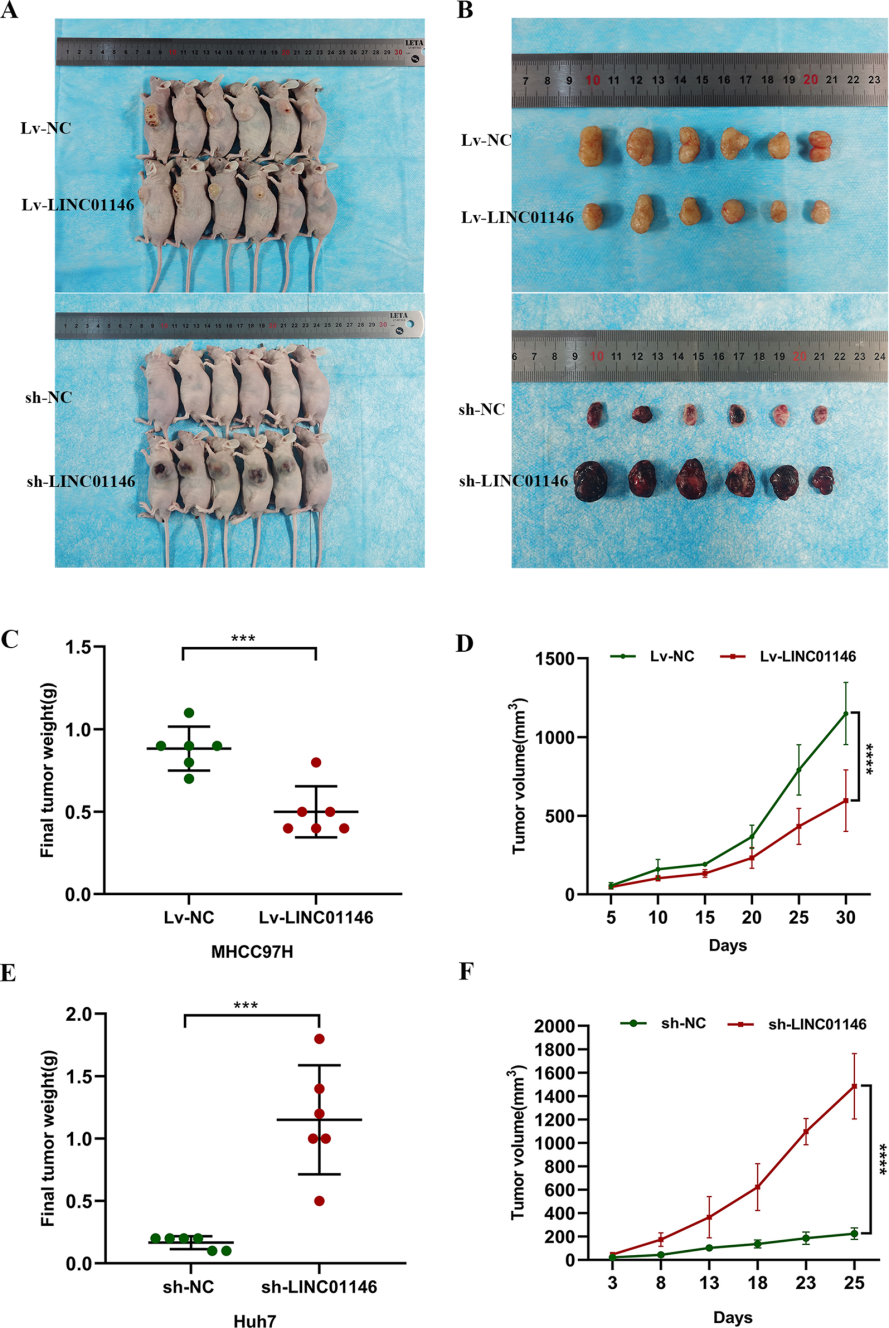
LINC01146 affects the tumour growth of HCC cells in vivo. **A**, **B** Construction of the tumor formation model in nude mice. **C**, **D** Overexpression of LINC01146 inhibited the tumour growth of HCC cells in nude mice. **E**, **F** Downregulation of LINC01146 promoted the tumour growth of HCC cells in nude mice. ***P < 0.01; ****P < 0.001

The tumour formation model in nude mice was used to evaluate the effect of LINC01146 on the proliferation of HCC cells in vivo (Fig. 6A, B). The results showed that the weight (*P* = 0.001, Fig. 6C) and volume (*P* < 0.001, Fig. 6D) of tumours were significantly smaller in the overexpressed group, while tumours (weight: *P* = 0.001, Fig. 6E; volume: *P* < 0.001, Fig. 6F) were significantly larger in the downregulated group than in the control group.

**Fig. 7:**
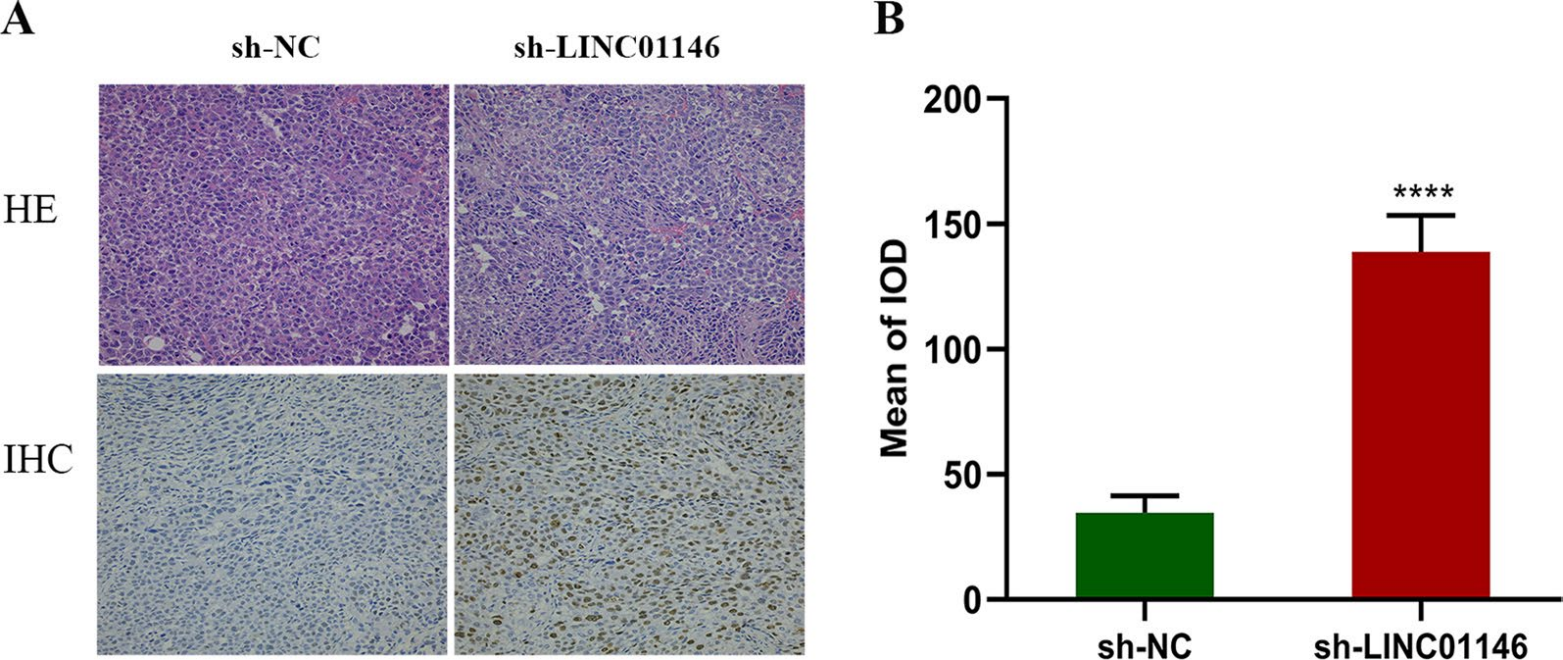
Downregulation of LINC01146 promotes the tumour growth of HCC cells in vivo. **A** The results of HE and IHC staining of tumour tissues of nude mice. **B** Downregulation of LINC01146 promoted the positive expression of Ki-67 protein. ****P < 0.001

HE staining was used to detect the effect of LINC01146 on the morphology and structure of HCC. As shown in Fig. 7A (HE), the tumour cells in the LINC01146 downregulation group were irregular in morphology and different in nuclear size and shape, with obvious atypia, deep staining, shrinkage of nuclear membrane, active mitosis, and pathological mitosis compared with the control group.

IHC staining was used to compare the expression levels of the cell proliferation marker protein Ki-67 between the LINC01146 downregulation group and the control group. The results showed that Ki-67 expression was significantly higher in the LINC01146 downregulation group than in the control group (*P* < 0.001; Figs. 7A (IHC), B), which further indicates that downregulation of LINC01146 promotes the growth of HCC cells in vivo.

### Functional and pathway enrichment analysis

**Fig. 8:**
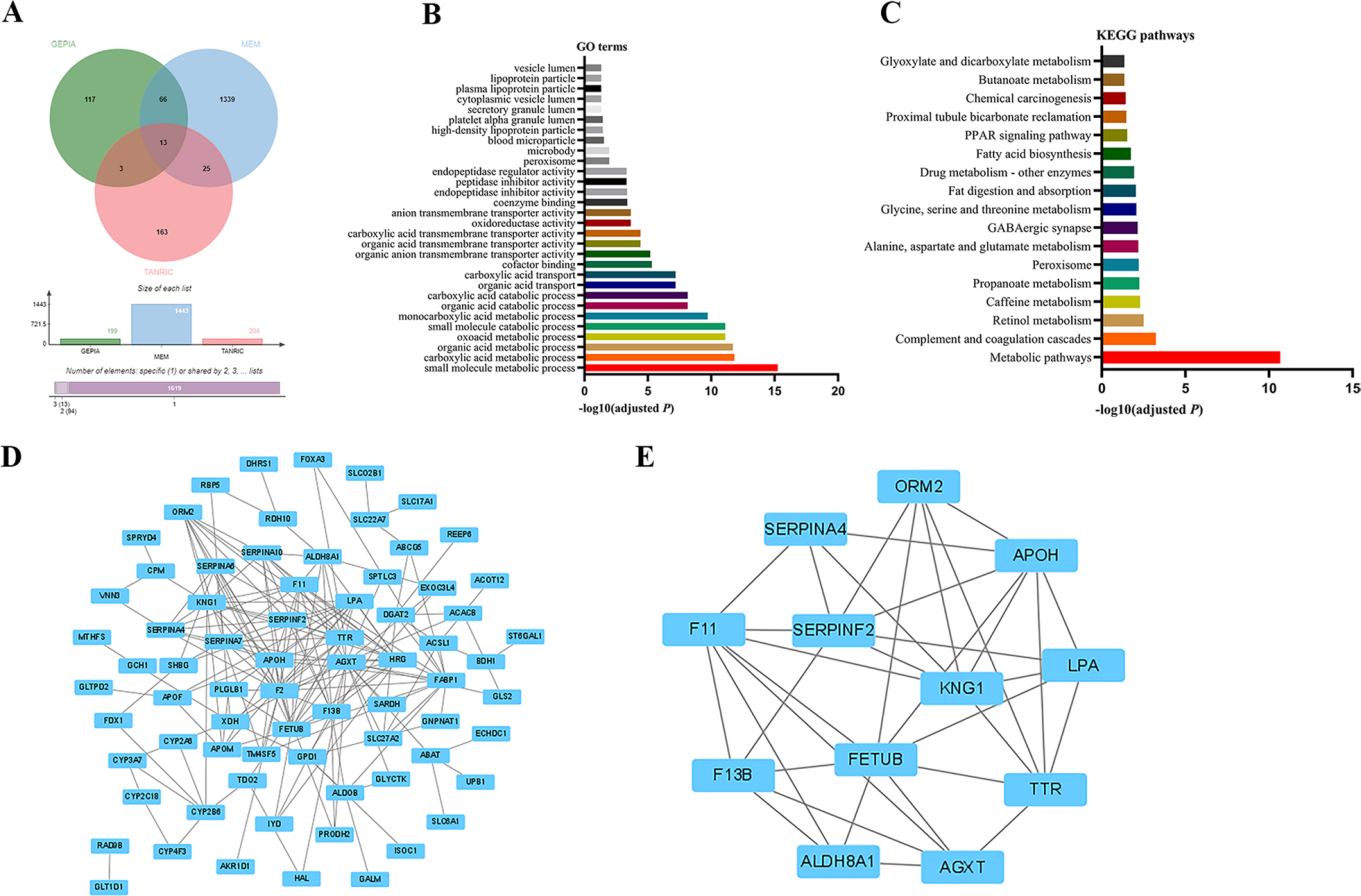
Pathway enrichment analyses and PPI network of LINC01146 in HCC. **A** A Venn diagram was used to identify intersecting genes using the GEPIA, MEM, and TANRIC websites. **B** Significant GO terms were enriched using the WebGestalt website. **C** Significant KEGG pathways were identified by the KOBAS 3.0 website. **D**, **E** The PPI network and core genes of the coexpressed genes were constructed by Cytoscape software

The GEPIA, MEM, and TANRIC websites were utilized to screen for promising coexpressed genes related to LINC01146. Specifically, 199, 1443, and 204 potential coexpressed genes were found on the GEPIA, MEM, and TANRIC websites, respectively, and 107 overlapping coexpressed genes were subsequently screened out for deeper functional and pathway enrichment analyses (Fig. 8A). The results of GO enrichment analysis indicated that these overlapping coexpressed genes were mainly involved in “small molecule metabolism”, “carboxylic acid metabolism”, “organic acid metabolism”, “ketoacid metabolism”, “small molecule decomposition” and other biological processes (Fig. 8B). The KEGG pathway enrichment analysis showed that these genes were mainly enriched in “metabolic pathway”, “complement and coagulation cascade”, “retinol metabolism”, “caffeine metabolism”, “propionic acid metabolism” and other pathways (Fig. 8C).

### Construction of the PPI network and identification of core genes

The STRING online website was used to construct a PPI network of 107 overlapping coexpressed genes to reveal their mutual associations. The results showed that the network has a total of 100 nodes and 181 edges, with an average node degree of 3.62 and an average local clustering coefficient of 0.37 (*P* < 1.0E−16, Fig. 8D). Subsequently, we used the MCODE plug-in of Cytoscape software to screen for core genes. The results showed that *FETUB* (Fetuin-B), *TTR* (transthyretin), *LPA* [lipoprotein(a)], *ALDH8A1* (aldehyde dehydrogenase 8 family member), *SERPINA4* (serpin family A member 4), *AGXT* (alanine–glyoxylate and serine–pyruvate), *APOH* (apolipoprotein H), *SERPINF2* (Serpin family F member 2), *F13B* (Coagulation factor XIII B chain), *ORM2* (Orosomucoid 2), *KNG1* (kininogen 1), and *F11* (Coagulation factor XI) were the 12 most significant core genes (Fig. 8E).

In addition, we explored the expression levels of these 12 core genes in HCC tissues based on the TCGA database and found that they were all lower in HCC tissues than in adjacent normal tissues (all *P* < 0.001, Table 4). Pearson correlation analysis was used to explore the correlation between LINC01146 and these 12 core genes. The results indicated that LINC001146 had the strongest correlation with *FETUB* (*P* < 0.001) and *TTR* (*P* < 0.001) among these 12 core genes, and the details are shown in Table 4.

**Table 4:** The expression levels of core genes in HCC tissues and the correlation between core genes and LINC01146

Core genes	Tissue types	N	Expression (χ ± s)	*t*	*P* value	Pearson correlation	
						*r*	*P* value
FETUB	HCC tissue	371	5.45 ± 2.40	− 12.00	< 0.001	0.56	< 0.001
	Normal tissue	50	7.28 ± 0.62				
TTR	HCC tissue	371	10.14 ± 2.78	− 11.83	< 0.001	0.53	< 0.001
	Normal tissue	50	11.93 ± 0.76				
LPA	HCC tissue	371	2.69 ± 1.37	− 17.29	< 0.001	0.49	< 0.001
	Normal tissue	50	4.83 ± 0.72				
ALDH8A1	HCC tissue	371	5.49 ± 1.66	− 18.96	< 0.001	0.46	< 0.001
	Normal tissue	50	7.33 ± 0.32				
SERPINA4	HCC tissue	371	7.26 ± 1.89	− 8.81	< 0.001	0.46	< 0.001
	Normal tissue	50	8.37 ± 0.57				
AGXT	HCC tissue	371	8.56 ± 2.08	− 11.13	< 0.001	0.45	< 0.001
	Normal tissue	50	9.88 ± 0.34				
APOH	HCC tissue	371	12.29 ± 1.98	− 8.09	< 0.001	0.44	< 0.001
	Normal tissue	50	13.20 ± 0.32				
SERPINF2	HCC tissue	371	9.72 ± 1.62	− 11.53	< 0.001	0.43	< 0.001
	Normal tissue	50	10.78 ± 0.27				
F13B	HCC tissue	371	6.26 ± 1.75	− 3.53	< 0.001	0.41	< 0.001
	Normal tissue	50	6.65 ± 0.45				
ORM2	HCC tissue	371	10.51 ± 1.69	− 11.97	< 0.001	0.38	< 0.001
	Normal tissue	50	11.96 ± 1.69				
KNG1	HCC tissue	371	10.00 ± 1.89	− 4.08	< 0.001	0.37	< 0.001
	Normal tissue	50	10.44 ± 0.32				
F11	HCC tissue	371	5.01 ± 1.34	− 10.20	< 0.001	0.32	< 0.001
	Normal tissue	50	5.97 ± 0.45				

## Discussion

In the present study, we identified LINC01146 as a novel liver-specific lncRNA. We showed that LINC01146 was decreased in HCC tissues and expression of LINC01146 was negatively related to the aggressive clinical features and poor prognosis of HCC patients. Furthermore, overexpression of LINC01146 inhibited the proliferation, migration, and invasion while promoting the apoptosis of HCC cells in vitro. In contrast, downregulation of LINC01146 exerted the opposite effects. Moreover, overexpression of LINC01146 inhibited the tumour growth of HCC cells in vivo, while downregulation of LINC01146 played the opposite role in vivo. All of the above results indicated that LINC01146 may play a cancer-inhibiting role in HCC progression.

HCC patients with aggressive clinical features are prone to recurrence, metastasis, and poor prognosis. Previous studies have shown that MVI indicates the metastasis of HCC, and patients with positive MVI have a poor prognosis [35, 36]. The increase in tumour size is one of the signals of metastasis in HCC patients and is directly proportional to the risk of metastasis in HCC patients [37]. Additionally, the presence of satellite nodules in the pathological diagnosis of HCC patients shows invasion of cancer cells and suggests poor prognosis of HCC patients and increased proneness to symptom recurrence [38, 39]. A great deal of evidence suggests that lncRNAs can serve as molecular targets to predict the prognosis of HCC patients, such as lncRNA DGCR5, lncRNA GAS5-AS1, lncRNA miR210Hg, and lncRNA SNHG16 [40–43]. In our study, we found for the first time that low expression of LINC01146 was associated with aggressive clinical features, including tumour size, tumour number, MVI, satellite nodules, DNA content of HBV, and BCLC grade. In addition, we found that low expression of LINC01146 was associated with poor prognosis in HCC patients. These results suggested that low expression of LINC01146 may affect the progression and prognosis of HCC patients and may serve as a tumour inhibitor and a prognostic molecular marker for HCC patients.

In the present study, we identified LINC01146 as a liver-specific lncRNA enriched in normal liver tissues based on the GTEx database and involved in the proliferation, migration, invasion, and apoptosis of HCC cells. Recently, more studies have shown that tissue-specific lncRNAs play an important role in the development of cancers. For instance, two tissue-specific lncRNAs, PCAT18 and LINC01133, were enriched in normal stomach tissues and downregulated in gastric cancer (GC) [44]. PCAT18 acts as a cancer inhibitor by impairing the viability, invasion, and migration of GC cells [45]. LINC01133 inhibits the proliferation, migration, and epithelial–mesenchymal transition (EMT) of GC cells by silencing the Wnt/β-catenin pathway [46]. LncRNA TINCR plays a tissue-specific role in normal skin, placenta, and oesophageal tissues [47]. Several studies have reported that TINCR plays a cancer suppressive role in lung cancer, breast cancer, and prostate cancer by inhibiting biological characteristics, such as proliferation, migration, and invasion of cancer cells [48–50]. These findings suggest that tissue-specific lncRNAs may play an inhibitory role in tumourigenesis.

The negative correlation between the expression of LINC01146, MVI, and satellite nodules prompted us to hypothesize that LINC01146 may be involved in the proliferation, migration, invasion, and apoptosis of HCC cells. Rapid proliferation is one of the most important biological characteristics of HCC cells [51]. LncRNAs can affect the proliferation of HCC cells by targeting key regulatory factors in different pathways. For instance, lncRNA MALAT1 promotes HCC cell proliferation by regulating expression of the oncogenic transcription factor B-MYB to facilitate cell cycle progression [52]. Overexpressed lncRNA SNHG16 sponges hsa-miR-93 and inhibits the proliferation of HCC cells [53]. The upregulation of lncRNA FAM83H-AS1 promotes HCC cell proliferation through the Wnt/β-catenin pathway [54]. Previous studies also revealed that HCC patients harbouring cancer cells with high migration and invasion activities often have increased aggressiveness, recurrence, and poor survival. For example, lncRNA MYLK-AS1 facilitates HCC progression and angiogenesis by promoting the invasion and metastatic abilities of HCC cells in vivo through targeting the miR-424-5p/E2F7 axis and activating the VEGFR-2 signalling pathway [55]. LncRNA HAND2-AS1 inhibits the proliferation, migration, and invasion of SNU-398 cells by mediating the downregulation of ROCK2 protein in HCC [56]. Silencing of LINC00240 suppresses the migration and invasion of HCC cells by promoting miR-4465 and inhibiting the HGF/c-Met signalling pathway [57]. In the present study, by overexpressing and knocking down LINC01146, we found that LINC01146 inhibited the proliferation, migration, and invasion abilities of HCC cells in vitro and promoted their apoptosis. Additionally, LINC01146 inhibited the growth of tumours in vivo. These results suggest that LINC01146 may play a cancer-inhibiting role in HCC by reducing biological characteristics of HCC cells, such as proliferation, migration, and invasion, and promoting apoptosis.

Previous studies have reported that lncRNAs influence the occurrence and progression of tumours by participating in a variety of pathways. We found that the coexpressed genes of LINC01146 were mainly involved in “metabolic pathway”, “complement and coagulation cascade”, “retinol metabolism”, “caffeine metabolism” and other pathways. The reprogramming of cell energy metabolism, which provides an energetic basis for the unrestricted proliferation and metastasis of cancer cells, is widely recognized as an emerging cancer marker. Metabolic reprogramming plays a key role in promoting tumour survival and proliferation to sustain the increasing metabolic demands of cancer cells [58, 59]. In recent years, complement and coagulation cascades have played crucial roles in the carcinogenesis and progression of cancer [60–62]. Complement cascades contribute to the development of the major features of carcinogenesis, including the maintenance of cell proliferation, inhibition of apoptosis, and promotion of cell invasion [63]. In addition, many bioinformatic studies have shown that retinol metabolism plays an important role in the occurrence and development of HCC [64–66]. Caffeine has also been reported to have an antitumour effect and can protect liver function. Edling et al. found that caffeine blocks the proliferation of HCC and pancreatic cancer adenocarcinoma cells by inhibiting the PI3K/Akt pathway [67]. Moreover, Okano et al. reported that caffeine inhibits the proliferation of HCC cells by activating the MEK/ERK/EGFR signalling pathway [68]. We also found a strong correlation between the expression levels of *FETUB*/*TTR* and LINC01146 in HCC. However, we only discovered this relationship through the TCGA database and did not verify it through experimental methods, which requires further study.

In conclusion, LINC01146 is downregulated in HCC tissues and negatively correlated with aggressive clinical features and poor prognosis of HCC patients. It may serve as a prognostic biomarker for HCC patients and a cancer suppressor by repressing the proliferation, migration, and invasion abilities of HCC cells, while promoting the apoptosis of HCC cells.

## Supplementary Information

**Additional file 1: Figure S1.** The supplementary materials for LINC01146. **A** Volcano map of differentially expressed lncRNAs in the GSE93789 microarray dataset. **B** LINC01146 was specifically expressed in normal liver tissues. **C** No apparent publication bias was observed in this meta-analysis by funnel plot. **D** The results of this meta-analysis were stable by sensitivity analysis.

**Additional file 2: Table S1.** The expression of LINC01146 in GSE93789 microarray.

## Data Availability

The datasets used and/or analyzed during the current study are available from the corresponding author on reasonable request.
