# Supplementary figures and images for: Liver-specific LINC01146, a promising prognostic indicator, inhibits the malignant phenotype of hepatocellular carcinoma cells both in vitro and in vivo
Source: J Transl Med. 2022 Jan 31;20:57. doi: 10.1186/s12967-021-03225-2 (PMC8802422; doi:10.1186/s12967-021-03225-2)

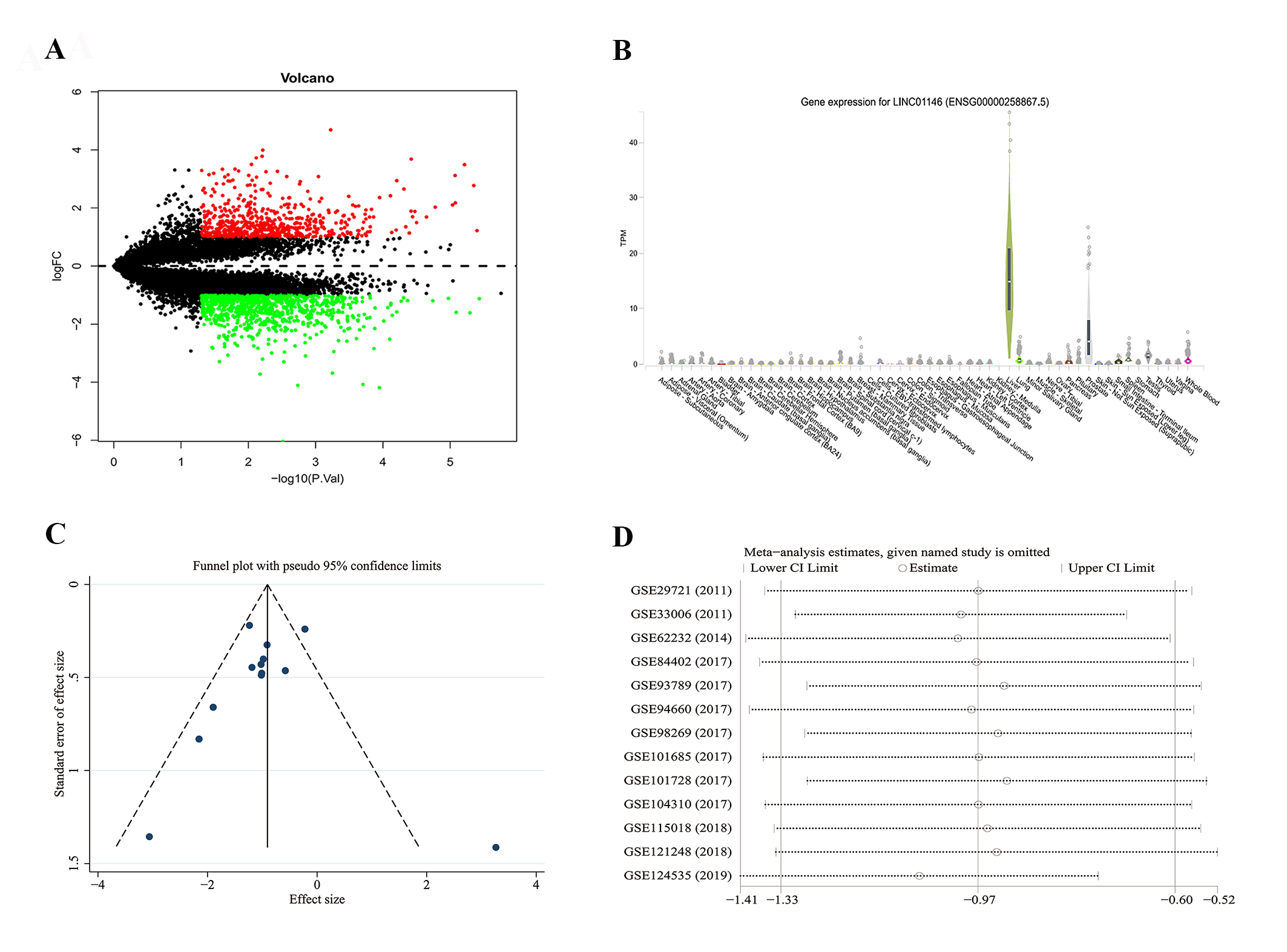

Supplement: Supplementary file 1 — Additional file 1: Figure S1. The supplementary materials for LINC01146. A Volcano map of differentially expressed lncRNAs in the GSE93789 microarray dataset. B LINC01146 was specifically expressed in normal liver tissues. C No apparent publication bias was observed in this meta-analysis by funnel plot. D The results of this meta-analysis were stable by sensitivity analysis. [file 12967_2021_3225_MOESM1_ESM.tif]
