# Supplementary material for: Liver-specific LINC01146, a promising prognostic indicator, inhibits the malignant phenotype of hepatocellular carcinoma cells both in vitro and in vivo
Source: J Transl Med. 2022 Jan 31;20:57. doi: 10.1186/s12967-021-03225-2 (PMC8802422; doi:10.1186/s12967-021-03225-2)
Supplement: Supplementary file 2 — Additional file 2: Table S1. The expression of LINC01146 in GSE93789 microarray. [file 12967_2021_3225_MOESM2_ESM.doc]

| **Table S1 The expression of LINC01146 in the lncRNA microarray GSE93789** | | | | | |
| --- | --- | --- | --- | --- | --- |
| **Gene symbol** | ***P* value** | **FDR** | **Fold Change** | **Regulation** | **Probe name** |
| LINC01146 | 6.93E-04 | 0.027 | 3.92 | downregulation | ASHGA5P022257 |
